# Quantile regression for genome-wide association study of flowering time-related traits in common bean

**DOI:** 10.1371/journal.pone.0190303

**Published:** 2018-01-04

**Authors:** Moysés Nascimento, Ana Carolina Campana Nascimento, Fabyano Fonseca e Silva, Leiri Daiane Barili, Naine Martins do Vale, José Eustáquio Carneiro, Cosme Damião Cruz, Pedro Crescêncio Souza Carneiro, Nick Vergara Lopes Serão

**Affiliations:** 1 Department of Statistics, Federal University of Viçosa, Viçosa, Minas Gerais, Brazil; 2 Department of Animal Science, North Carolina State University, Raleigh, North Carolina, United States of America; 3 Department of Animal Science, Federal University of Viçosa, Viçosa, Minas Gerais, Brazil; 4 Department of Plant Sciences, Federal University of Viçosa, Viçosa, Minas Gerais, Brazil; 5 Department of General Biology, Federal University of Viçosa, Viçosa, Minas Gerais, Brazil; 6 Department of Animal Science, Iowa State University, Ames, Iowa, United States of America; Huazhong University of Science and Technology, CHINA

## Abstract

Flowering is an important agronomic trait. Quantile regression (QR) can be used to fit models for all portions of a probability distribution. In Genome-wide association studies (GWAS), QR can estimate SNP (Single Nucleotide Polymorphism) effects on each quantile of interest. The objectives of this study were to estimate genetic parameters and to use QR to identify genomic regions for phenological traits (Days to first flower—DFF; Days for flowering—DTF; Days to end of flowering—DEF) in common bean. A total of 80 genotypes of common beans, with 3 replicates were raised at 4 locations and seasons. Plants were genotyped for 384 SNPs. Traditional single-SNP and 9 QR models, ranging from equally spaced quantiles (τ) 0.1 to 0.9, were used to associate SNPs to phenotype. Heritabilities were moderate high, ranging from 0.32 to 0.58. Genetic and phenotypic correlations were all high, averaging 0.66 and 0.98, respectively. Traditional single-SNP GWAS model was not able to find any SNP-trait association. On the other hand, when using QR methodology considering one extreme quantile (τ = 0.1) we found, respectively 1 and 7, significant SNPs associated for DFF and DTF. Significant SNPs were found on Pv01, Pv02, Pv03, Pv07, Pv10 and Pv11 chromosomes. We investigated potential candidate genes in the region around these significant SNPs. Three genes involved in the flowering pathways were identified, including *Phvul*.*001G214500*, *Phvul*.*007G229300* and *Phvul*.*010G142900*.*1* on Pv01, Pv07 and Pv10, respectively. These results indicate that GWAS-based QR was able to enhance the understanding on genetic architecture of phenological traits (DFF and DTF) in common bean.

## Introduction

The common bean (*Phaseolus vulgaris L*.) is the world’s most important grain legume for human consumption [1]. Common bean is cultivated especially in African and Latin American countries, and is a key commodity for food security improvement because of the low-input agricultural systems required for production [2]. Phenological traits associated with the flowering time, such as days to flowering (DTF), days to first flower (DFF), and days to end of flowering (DEF), are used in selection programs of the common bean. Cultivars having early life-cycles can, reduce water consumption of irrigated crops, can quickly free the area for crop succession, can be beneficial in periods of less rain, and has lower time to be exposed to plagues and diseases [3].

Several genetic mapping studies have been performed for the common bean, improving the understanding the genetic architecture of relevant traits [4, 5, 6, 7]. However, due to the limited number of markers and small population sizes, these studies resulted in low-resolution QTL identification [2]. Thus, the inferences on positional candidate genes associated with the identified QTL have been widely poor.

In human genetics, Single Nucleotide Polymorphism (SNP) effects were estimated to be related to particular quantiles (extreme tails) on the distribution of obesity-related traits [8]. The authors demonstrated, based on gene function, that this strategy resulted in the detection of true QTL in genome-wide association studies (GWAS). The sampling from the extremes has been successfully used [9, 10] and can often to improve the resolution QTL identification [11]. The statistical power of association analyses can be improved by preferential selection of individuals who are most likely to be informative [9]. The main idea is to sample individuals with extreme phenotypes in the hopes that rare causal variants will be enriched among them. However, the same authors list some limitations of extreme phenotype sampling. Among them, the results might not be generalizable to the underlying population and might be sensitive to outliers, sampling bias, and the assumption of normality for the underlying traits [9].

An interesting approach to address this issue is the use of quantile regression (QR; [12]). Unlike traditional regression methods, which are based on conditional expectations, in other words E(X|Y), QR is based on conditional quantiles, Qτ(Y|X). Thus, QR approach can be used in all situations that traditional regression methods are applied, but gives a more complete picture of phenomenon in study. By using QR, it is possible to fit models to all portions of a probability distribution of the trait. In other words, QR enables to measure the impact of a SNP on specific quantiles of the trait. This feature takes into account the extreme tails of phenotypic distribution without sampling these parts specifically, that is, to sample individuals with extreme phenotypes. Therefore, QR uses the same principle used in sampling from the extremes of the trait distribution, except that with QR, estimation is performed directly on the extremes and a specific sampling is not needed, avoiding sampling bias [13].

QR have been successfully applied to GWAS in human genetics [14] and animal breeding [15]. In both, the principle of estimating markers effects under different quantiles provided more informative results than those based on traditional regression methods. However, to the best of our knowledge, there are no reports in literature approaching QR for plant breeding under a GWAS framework.

In this context, we aimed to (1) estimate genetic parameters for phenological traits in the common bean; (2) to enhance the understanding of the genetic architecture of these traits through quantile regression-based GWAS; and (3) to annotate candidate genes through the identification of the significant SNP positions at different quantiles.

## Materials and methods

### Flowering traits

Eighty common bean cultivars widely studied between 1970 and 2013 by several research institutions in Brazil (Embrapa, IAC, UFV, IAPAR, Epamig, UFLA, Fepagro, Epagri, FT seeds) were evaluated. These cultivars were selected based on scientific records, as well as on reports of breeders participating in different breeding programs [16]. The list of cultivars and research institutions is presented in S1 Table.

These cultivars were field planted at two experimental stations at the Federal University of Viçosa (*Universidade Federal de Viçosa*; UFV, Brazil), one located in Viçosa, MG, and the other in Coimbra, MG. Cultivars were planted at each location in the dry (February) and winter (July) seasons of 2013, following a randomized complete block design with three replicates. Each cultivar was planted in plots of four lines 3.0 m and interrow spacing of 0.5 m. Phenological traits for days to first flower (DFF), days to flowering (DTF), and days to end of flowering (DEF) were collected on all seasons and locations. DFF was measured as the number of days from planting until at least one plant presented a flower. DTF was measured as the number of days from planting to when at least 50% of the plants in a plot (replicate) had at least one open flower. DEF was measured as the number of days from planting to the time the all plants in the plot stopped flowering.

### Genotypic data

DNA samples were genotyped using the Vera Code1 BeadXpress platform (Illumina) at the Biotechnology Laboratory of Embrapa (Goiania, GO, Brazil). A set of 384 SNP markers, validated by a previously identified Prelim file (https://icom.illumina.com/Custom/UploadOpaPrelim/) for *Phaseolus vulgaris*, was selected to compose the Oligo Pool Assay (OPA) SNP marker panel. During the procedure for SNP detection, three oligonucleotides were used for each of the variants of the same SNP and the third specific-locus binding to the 3’ region of the DNA fragment containing the target SNP, generating a unique allele-specific fragment. Genotype call was performed using Genome Studio software version 1.8.4 (Illumina, City, State, USA), with Call Rate values ranging from 0.80 to 0.90 and GenTrain ≥ 0.26 for SNP clustering. Analyses were performed to cluster the SNP alleles of each line, based on signal intensities of Cy3 and Cy5 fluorophores. The phenotypic and genotypic data sets are freely accessible at https://zenodo.org/record/1120366#.WjqyZkxFzmQ.

### Data analysis

The phenotypic data for each phenologycal traits (DFF, DTF and DEF), were analyzed according to the following statistical model:

$$y_{ijk}=\mu {+g}_{i}+a_{j}+{ga}_{ij}++b_{k(j)}+\varepsilon_{ijk},$$

where y_ijk_ is the observed phenotype; μ is the general mean; g_i_ the random effect of genotype (cultivar) i, with i = 1 to 80; a_j_ is the fixed effect of environment j, with j = 1 to 4; ga_ij_ the random effect of the interaction of genotype i with environment j; b_k(j)_ is the random effect of block (replicate) k (k = 1, 2, 3) within environment j; and ε_ijk_ is the experimental error, Normally and Independently Distributed (NID).

Models with and without the interaction between genotypes and environment were compared using the Akaike information criterion (AIC) and Bayesian information criterion (BIC) were used to compare and select the best model. After selection of the model for each trait, genetic parameters (heritability and correlations) were estimated for lowering traits. In addition, adjusted phenotypes (Y*) were calculated as the sum of random effects in the model and used for association analyses. Analyses were carried out in ASReml 3.0 [17].

### Genome-wide association study

Prior to performing genome-wide association (GWA) analyses, the genetic structure of the populations used in this study was assessed by principal component analysis (PCA) using the *princomp* function in R [18]. PCA was applied to genotype data to obtain the top principal components (PC) that captured most of the genetic variation based on visual inspection of the scree plot. After that, the selected PC were used as covariates in the GWA models to detect SNPs associated with the phenologycal traits (DFF, DTF and and DEF). The general GWA model was given by:

$${\text{Y}}_{\text{ijk}}*=\mu +{\text{SNP}}_{\text{i}}+\sum_{\text{k}=1}^{7}{\text{PC}}_{\text{jk}}+\varepsilon_{\text{ijk}},$$

where Y_ijk_* is the adjusted phenotype; μ is the population mean; SNP_i_ is the fixed effect of the i^th^ SNP, fitted as a covariate (allele-substitution effect); PC_kj_ is the fixed effect (covariate) of k^th^ principal component for individual k; and ε_ijk_ is the random error term associated with Y_ijk_*. The vector **θ** = [μ, SNP_*i*_, *PC*_*jk*_]^T^ represents the unknown parameters.

The SNP effects were estimated at different portions of a probability distribution of the traits by quantile regression [12]. This method consists in estimating the parameters at quantile τ(**θ**_τ_) by solving the following optimization problem:

$${\hat{\theta }}_{\tau }={\text{argmin}}_{{\hat{\theta }}_{\tau }}\left[\overset{80}{\underset{\text{i}=1}{\sum }}\rho_{\tau }\left(\varepsilon_{\text{i}}\right)\right]$$

where τ indicates the quantile of interest. In the current study, we evaluated 9 quantiles (τ): 0.1 to 0.9, every 0.1. The parameter ρ_τ_(·) is denoted as a check function [12], and is defined by:

ρτ(εi)={τ∙εi,ifεi≥0,-(1-τ)∙εi,ifεi<0.

where τ ∈] 0,1 [ indicates the quantile of interest.

The markers effects were also obtained using the traditional GWA approach, by fitting one SNP at a time (allele-substitution effect). Thresholds (-log_10_P) of 3.88 and 4.58 were calculated based on the Bonferroni method for multiple testing to identify SNPs significant at 5% and 1%, respectively.

### Functional annotation

We exploited the potential biological mechanism of flowering traits by identifying genes close to the significant SNPs identified in this study using the Jbrowse on Phytozome v11.0 [19] for the common bean genome v1.0 [20]. Lists of genes located nearest to the significant SNP were extracted, allowing for a maximum distance of 3.5 Mb between SNP and annotated genes. For genes without clear functional annotation related to flowering traits, the genomic sequence data from Phytozome v11.0 was used in a search against NCBI database using BLASTn [21] with the objective of to finding functional annotations considering similar nucleotide sequences available on GenBank.

## Results

A summary including the means, standard deviations (SD) and ranges of the phenological traits are presented in Table 1.

**Table 1:** Summary statistics for phenological traits in the common bean.

Trait	Mean (SD)	Minimum	Maximum
DFF	34.7 (5.5)	20.0	49.0
DTF	42.3 (5.6)	27.0	55.0
DEF	53.0 (7.5)	34.0	71.0

DFF: Days to first flower; DTF: Days to flowering; DEF: Days to end of flowering.

### Model selection and genetic parameters

The goodness-of-fit for models with and without genotype-by-environmental interaction was assessed by AIC, BIC, and likelihood ratio test (LRT; Table 2). Overall, the full model (with the interaction effect) showed lower AIC and BIC values compared to the reduced model (without the interaction). For DFF, AIC and BIC were 2206 and 2225, respectively, for the full model and 2233 and 2248, respectively, for the reduced model. For DTF, these were 2318 (AIC) and 2338 (BIC), and 2333 (AIC) and 2348 (BIC), for the full and reduced models, respectively. For DEF, we obtained 2351 (AIC) and 2370 (BIC), and 2438 (AIC) and 2453 (BIC), for the full and reduced models, respectively. Finally, the LR test between full and reduced models were significantly different (*P*<0.01) for all 3 traits (Table 2).

**Table 2:** Goodness-of-fit measures for models with (Full) and without (Reduced) genotype-by-environmental interaction.

Trait^1^	Model	Goodness-of-fit^2^		
		AIC	BIC	LRT (P-Value)
DFF	Full	2206	2225	29.8 (<0.01)
	Reduced	2233	2248	
DTF	Full	2318	2337	17.26 (<0.01)
	Reduced	2334	2348	
DEF	Full	2351	2370	89.16 (<0.01)
	Reduced	2438	2453	

^1^DFF: Days to first flower; DTF: Days to flowering; DEF: Days to end of flowering.

^2^AIC, BIC, and likelihood ratio test–(LRT)

Genetic parameters are presented in Table 3. Estimates of heritability for DTF, DFF, and DEF were moderate to high, with 0.49±0.08, 0.58±0.06 and 0.32±0.05, respectively. Genetic and phenotypic correlation estimates were all positive and overall moderate to high. Between DFF with DTF and DEF, genetic correlations were 0.98±0.01 and 0.94±0.03, respectively, and phenotypic correlations were 0.68±0.04 and 0.83±0.02, respectively. Between DTF and DEF, correlations were 0.94±0.03 (genetic) and 0.66±0.04 (phenotypic).

**Table 3:** Genetic parameters^1^ (standard error)^2^ for phenological traits^3^ in the common bean.

**Trait**	**DFF**	**DTF**	**DEF**
DFF	0.58 (0.06)	0.68 (0.04)	0.83 (0.02)
DTF	0.98 (0.01)	0.49 (0.08)	0.66 (0.04)
DEF	0.94 (0.03)	0.94 (0.03)	0.32 (0.05)

^1^Heritability, and genetic and phenotypic correlations presented on the diagonal, lower off-diagonal, and upper off-diagonal, respectively.

^2^ The numbers in parentheses are the standard error of parameters estimates.

^3^DFF: Days to first flower; DTF: Days to flowering; DEF: Days to end of flowering.

### Population structure

The scree plot showing the explained variability for each principal component (PC), and the genotype dispersion based on the first two PCs are presented in S1 Fig. Analysis of population structure with PCA revealed that seven PCs accounted for ~82% of the genotypic variability (S1A Fig). These seven PCs were used as covariates in the GWA analyses to account for the population structure of the data. Although there was a tendency for some genotypes to cluster together in the upper left quadrant of the PCA plot using the first 2 PC (S1B Fig), there was no clear strong population structure observed.

### Genome-wide association

Association between SNPs and (adjusted) phenotypes were performed using traditional single-SNP and quantile regression (QR) methodologies. The traditional single-SNP approach did not result in any significant (*P*>0.1x10^-4^) associations between SNPs and phenological traits (DFF, DTF and DEF). The Manhattan plots for DFF, DTF and DEF are showed in S2–S4 Figs, respectively.

**Fig 1:**
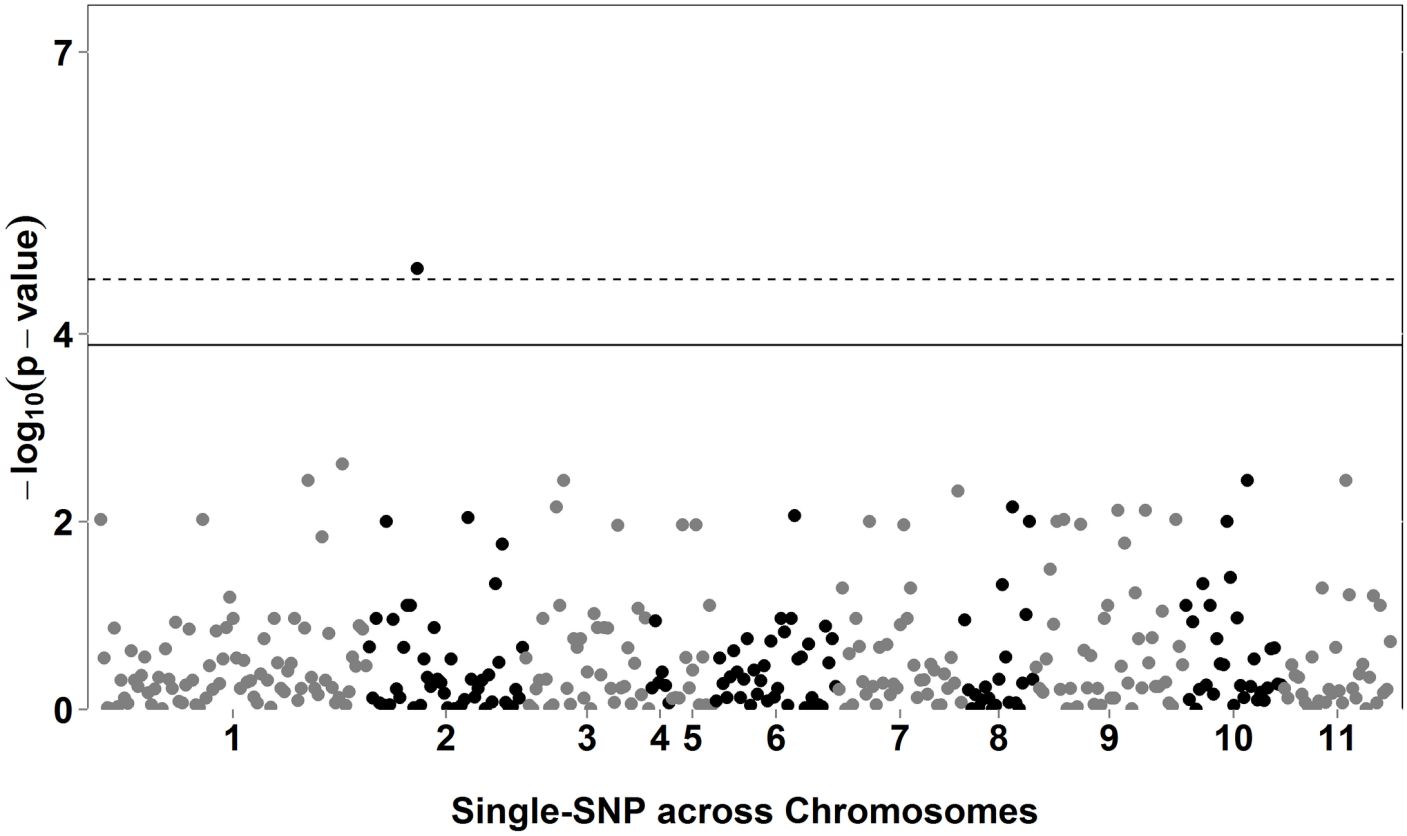
Manhattan plot for days to first flower (DFF) using quantile regression (QR) for quantile (*τ*) 0.1. The solid and dashed lines show the Bonferroni-adjusted thresholds of 3.88 and 4.58 for alpha equals to 5% and 1%, respectively.

**Fig 2:**
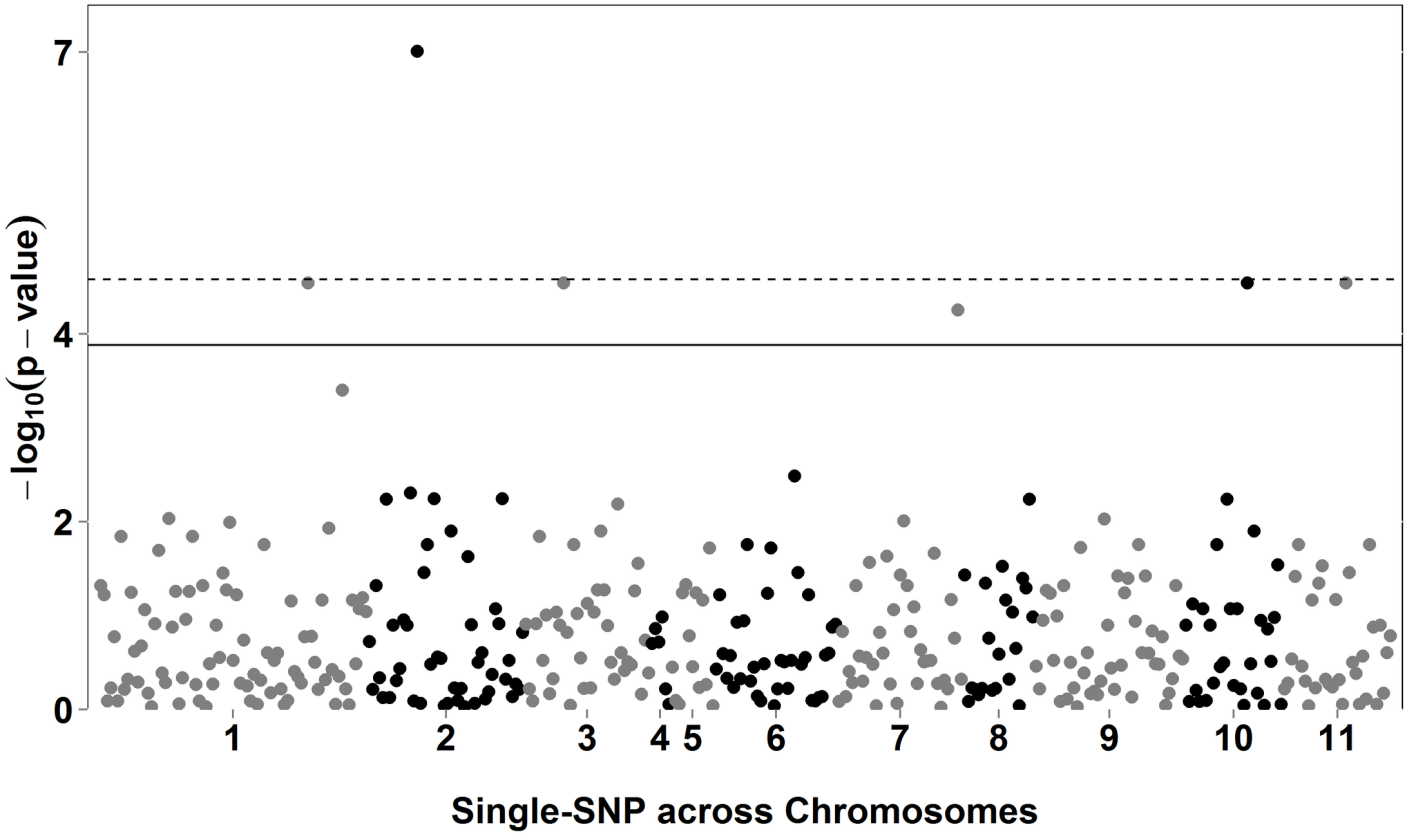
Manhattan plot for days to flowering (DTF), using quantile regression (QR) for quantile (*τ*) 0.1. The solid and dashed lines show the Bonferroni-adjusted thresholds of 3.88 and 4.58 for alpha equals to 5% and 1%, respectively.

**Fig 3:**
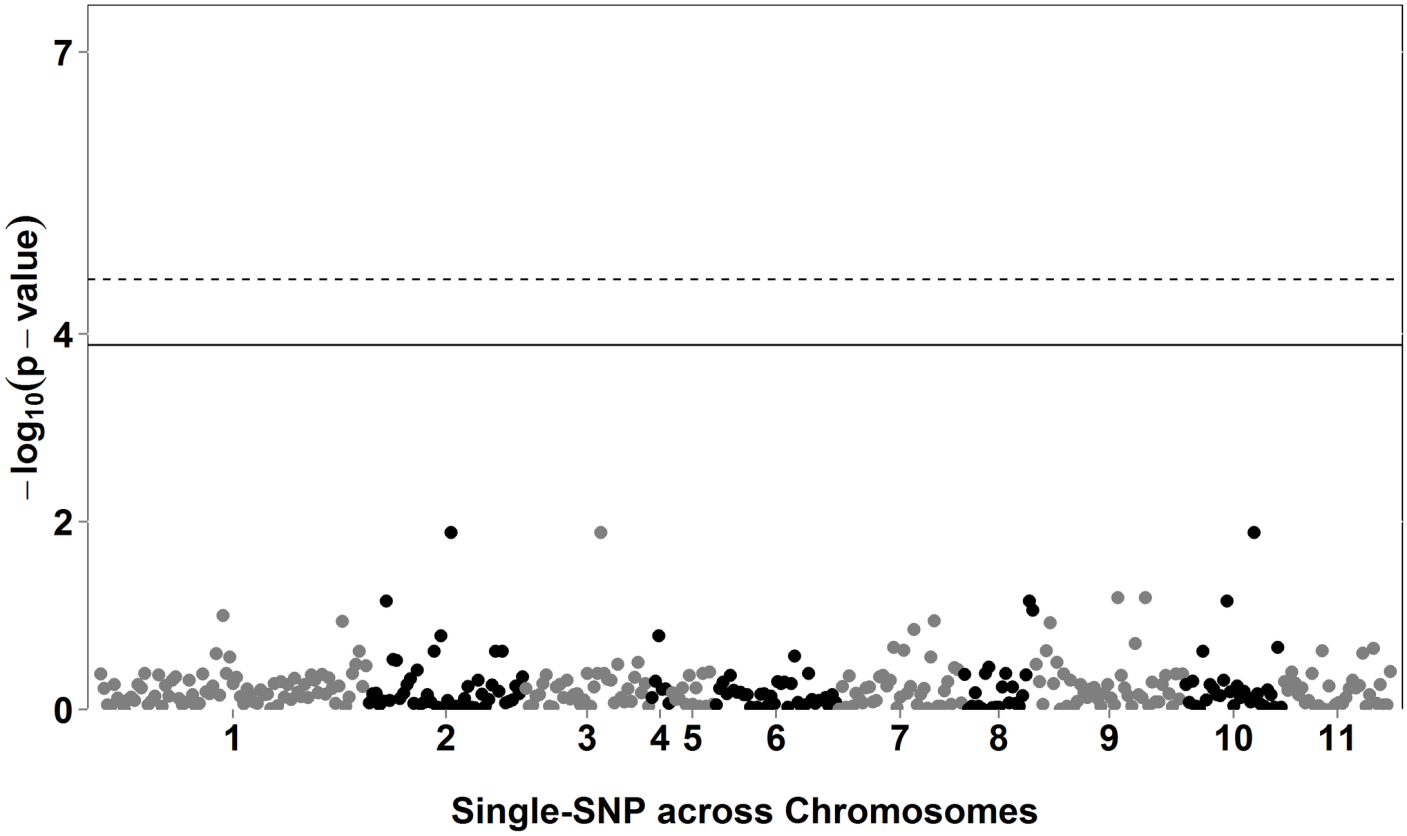
Manhattan plot for days to end of flowering (DEF), using quantile regression (QR) for quantile (*τ*) 0.1. The solid and dashed lines show the Bonferroni-adjusted thresholds of 3.88 and 4.58 for alpha equals to 5% and 1%, respectively.

Using the QR approach, which allows for the estimation of SNP effects at different portions of the probability distribution of the trait, associations were only found (*P*<0.1x10^-4^) using the lowest quantile (τ = 0.1) evaluated in this study (Table 4). We identified 1, 7, and no significant association (*P*<0.1x10^-4^) for DFF (Fig 1), DTF (Fig 2), and DEF (Fig 3), respectively. Manhattan plots using all quantiles (0.1 to 0.9) are presented in S5–S7 Figs, for DFF, DTF, and DEF, respectively.

**Table 4:** Single nucleotide polymorphisms (SNPs) associated with phenological traits in the common bean using quantile regression^1^, chromosome (Pv), position, *P*-values and proportion of phenotypic variation explained (R^2^) of most significant, using QR (τ = 0.1) model.

Trait^2^	SNP	Pv^3^	Position (Mb)	P-value	R^2^(%)^4^
DTF	scaffold00005_1715227	Pv01	50.33	2.88E-05	0.58
	scaffold00020_86873	Pv02	4.38	9.74E-08	0.41
	scaffold00083_261143	Pv03	11.79	2.88E-05	0.15
	scaffold00028_145636	Pv07	50.02	5.55E-05	1.97
	scaffold00036_329244	Pv10	41.67	2.88E-05	6.64
	scaffold00007_368524	Pv11	6.38	2.88E-05	2.30
DFF	scaffold00020_86873	Pv02	4.38	2.02E-05	1.92

^1^Using quantile (τ) 0.1;

^2^DFF: Days to first flower; DTF: Days to flowering; DEF: Days to end of flowering;

^3^*Phaseolus vulgaris* chromosome (Pv);

^4^R^2^, coefficient of determination.

**Fig 4:**
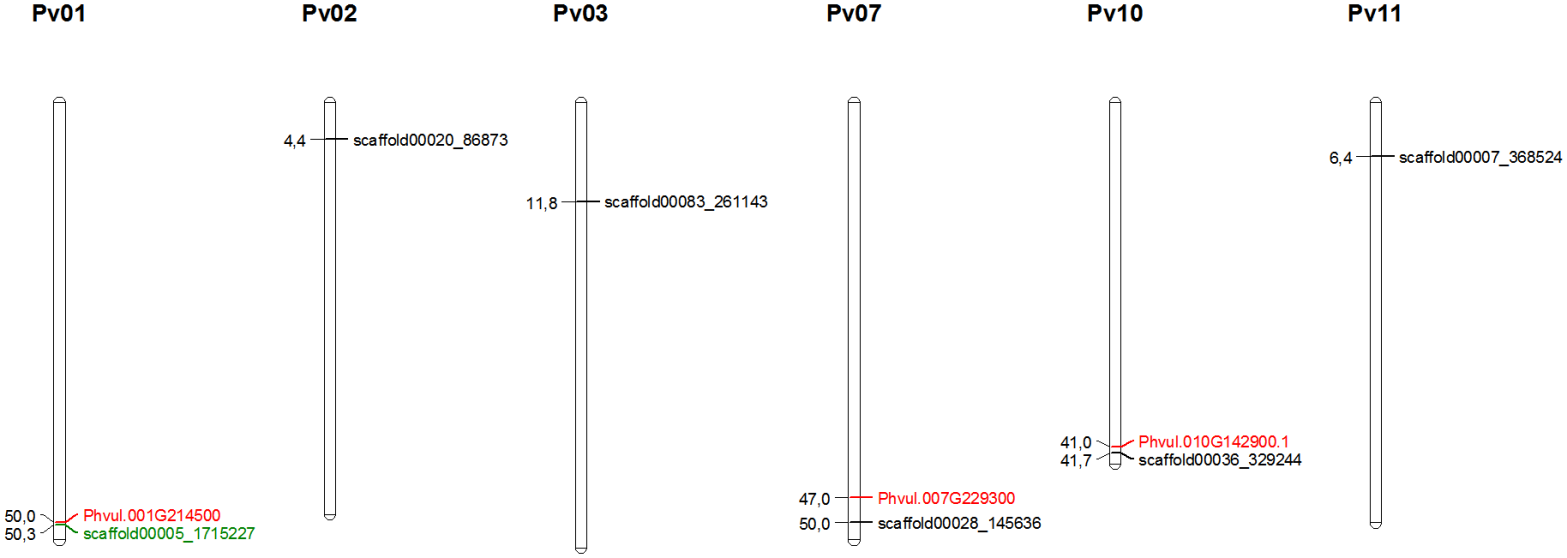
Representative map of the genome positions of markers associated (P<0.05) with DFF and DTF, and potential positional candidate genes. Pv: *Phaseolus vulgaris* chromosome. SNP markers were located based on the reference genome [20] using the Phytozome v11.0 BLAST tool (http://www.phytozome.net/). Markers in black are associated with DTF and those in green are associated with DTF and DFF jointly. Candidate genes are in red. The map was drawn with MapChart [38], and marker positions are represented in Mb.

The location of these SNPs are shown in Table 4 and depicted in Fig 4. For DTF, significant (*P* < 0.1x10^-4^) SNPs were identified on Pv01 (scaffold00005_1715227), Pv02 (scaffold00020_86873), Pv03 (scaffold00083_261143), Pv07 (scaffold00028_145636), Pv10 (scaffold00036_329244), and Pv11 (scaffold00007_368524). Altogether, these SNPs explained 12.5% of the variability in DTF (Table 3). One of the SNPs, scaffold00020_86873 on Pv02, was simultaneously associated with DTF and DFF. This SNP was the only significant association for DFF, and explained 1.92% of the variability in DFF and only 0.41% in DTF (*P* > 0.1x10^-4^).

After that, we investigated potential candidate genes in the region around these significant SNPs (Table 4). Three genes involved in the flowering pathways were identified, including Phvul.001G214500, Phvul.007G229300 and Phvul.010G142900.1 on Pv01, Pv07 and Pv10, respectively.

## Discussion

### Genetic parameters

Heritability estimates for DFF (0.58), DTF (0.49) and DEF (0.32) were consistent with those reported in the literature. Specifically, for DFF and DTF, estimates were close to those reported by [22], with 0.54 and 0.35 for DTF and DEF, respectively, and by [23], with 0.59 for DFF. As expected, the estimates of genetic and phenotypic correlations between the flowering time traits were positive and high (ranging from 0.66 to 0.98), which are similar those reported by [23] and [24], where values ranged from 0.82 to 0.90.

### Genome-wide association study

In this study, we aimed to enhance the understanding of the genetic architecture of phenological traits (DFF, DTF and DEF) in the common bean under a QR approach. The proposed method allows investigating the relationship between explanatory variables (i.e. SNPs in our study) and phenotype at different portions of a probability distribution of the traits, which resulted in more informative results by taking into account the extreme tails of trait distributions. QR uses the same principle used in sampling from the extremes of the trait distribution [8, 9, 10], except that estimation is performed directly on the extremes of the response variable, avoiding possible sampling biases [13].

Although the main objective of this manuscript was just to introduce the QR for plant breeders under a GWAS approach, we believe that this concept can be better exploited under a small sample size framework. The fact of QR take into account any quantile of phenotypic distribution can be considered as a robust tool to make inference in the presence of small population size. Although selective samples (sampling from the extremes) have been effective for QTL detection [8], under a small number of individuals, this method can implies in a drastic reduction of sample information because only the individuals belonging to the extremes will be considered for the analysis. Since QR uses all available information to infer on specific quantiles, this tool can be characterized as a robust method for small sample sizes. Tarr [25] and Ismail [26] studied the behavior of QR with small sample sizes and concluded about the robustness of this method under the mentioned situation. In terms of the small number of markers used here, it is interesting to highlight that the probability to identify significant markers is directly dependent of the genome coverage. In this context, the QR is a practical and efficient way to increase this probability since the same marker can be tested under different quantiles.

The number of reports including GWAS for economically relevant traits in the common bean is still very limited. Kamfwa et al. [2], using 237 genotypes of common beans genotyped for 5398 SNPs, found two significant SNPs for phenological traits DTF and days to maturity. These authors suggested the positional gene *Phvul*.*001G221100*, located on Pv01, as a candidate gene for controlling photoperiod sensitivity and flowering in common bean. Moghaddam et al. [22] observed significant SNP association on Pv01 for DTF, and on Pv04 and Pv11 for DTM, using over 150K SNPs in the analysis of 280 common bean genotypes. In terms of candidate genes, these authors listed 6 genes, for example, *Phvul*.*001G064600*, *Phvul*.*001G192200*, *Phvul*.*001G192300*, and *Phvul*.*001G087900* with potential role on days to flowering, because of their function on regulates Flowering Time [27]; change the expression periodicity of genes leading to earlier flowering [28]; suppressing the gibberellic acid pathway and directly interacts with GIGANTEA (GI), a gene within the photoperiod pathway [29]; regulates the photoperiod and vernalization pathway by directly interacting with and suppressing FLC [30], respectively.

In the present study, no associations were found when analyses were performed using traditional GWA (single-SNP GWAS) methodology. The lack of associations using this methodology was expected because of the limited power of this study, which is limited due to the small number of markers and population sizes available [2]. However, using the same principle used in sampling from the extremes by QR approach, that is, performing the estimation directly on the extremes, we found a total of 7 associations between SNPs and phenological traits using QR methodology: 6 for DTF and 1 for DFF. These associations were found considering an extreme quantile, more specifically, τ = 0.1. QTL reported here were previously identified on Pv01 [6, 31, 32, 33], Pv02 [33, 34], Pv07 [6, 31, 34] and Pv11 [31, 34]. Since previous studies have reported QTLs on the same regions, potential positional candidate genes for flowering traits around significant SNPs were investigated in our study.

Among the significant SNPs, 2 located on Pv01 and Pv07 are closest (<3.5Mb) to 2 genes involved in the flowering pathways. The first one, *Phvul*.*001G214500*, located on Pv01 (~50Mb). The functional annotation on Phytozome indicated that *Phvul*.*001G214500* is a *flowering time control protein FCA-related*. This protein plays a role in the regulation of flowering time in the autonomous flowering pathway by decreasing expression levels of *flowering locus C* [35]. According to Phytozome, *Phvul*.*007G229300*, which was located on Pv07 (~47Mb), is classified as *protein terminal flower 1*. A BLASTn search on NCBI data resulted in a best score to a gene TFL1z. Flowering time (FT) acts in part downstream of transcription factor gene, Constans (CO), and mediates signals for flowering in an antagonistic manner with the TFL1 gene. *Phvul*.*010G142900*.*1* that is mapping near the Pv10 (~41Mb) is a protein *early flowering 3* [36]. This protein participates in the initiation of flowering [37]. The representative map of the genome positions of markers associated with DFF and DTF and potential positional candidate genes for flowering in the region around significant SNPs are showed in Fig 4. For the other four associated markers, no genes related to flowering time-related traits were identified based on Phytozome.

In general, these results indicate that using quantile regression to perform GWAS for flowering traits in the common bean resulted in the identification of QTLs. In contrast, this was not the case for when we used traditional single-SNP analyses. Indeed, these results are reasonable, once QR allows the estimation of effects for all portions of a probability distribution of the trait, using of the same principle used in sampling from the extremes of the trait distribution, increase the statistical power of the analysis [8, 9, 10]. This improved power compared to traditional GWAS should be even greater with the increase of sample size and SNP density. Despite QR approach seems interesting in situations as the limited number of markers and small population sizes, more studies using simulated and real data sets with different genetic architectures and data set sizes (individuals and markers) are needed to confirm the efficiency of QR compared to traditional genome-wide association approaches.

## Conclusion

Quantile regression-based GWAS was able to detect true QTL for phenological traits (DFF and DTF) in the common bean that were missed when using traditinal single-SNP. The associations were identified using one extreme quantile (τ = 0.1), using the same principle used in sampling from the extremes. Finally, genomic regions identified in this study were enriched with candidate genes involved in the flowering pathways.

## Supporting information

S1 Table Name of cultivars, year of release and research institution in charge of the 80 common bean cultivars. (PDF)

S1 Fig Principal component (PC) analysis. (A) Scree plot and variance explained (%) by each component. (B) Plot for PC1 against PC2 illustrating the population structure of 80 common bean genotypes.
(TIFF)

S2 Fig Manhattan plot for days to first flower (DFF), using traditional single-SNP analysis. The solid and dashed lines show the Bonferroni-adjusted thresholds of 3.88 and 4.58 for alpha equals to 5% and 1%, respectively.
(TIFF)

S3 Fig Manhattan plot for days to flowering (DTF), using t traditional single-SNP analysis. The solid and dashed lines show the Bonferroni-adjusted thresholds of 3.88 and 4.58 for alpha equals to 5% and 1%, respectively.
(TIFF)

S4 Fig Manhattan plot for days to end of flowering (DEF), using traditional single-SNP analysis. The solid and dashed lines show the Bonferroni-adjusted thresholds of 3.88 and 4.58 for alpha equals to 5% and 1%, respectively.
(TIFF)

S5 Fig Manhattan plot for days to first flower (DFF) using quantile regression (QR). Results are sorted by quantile (τ), from 0.1 (A) to 0.9 (I). The solid and dashed lines show the Bonferroni-adjusted thresholds of 3.88 and 4.58 for alpha equals to 5% and 1%, respectively.
(TIFF)

S6 Fig Manhattan plot for days to flowering (DTF) using quantile regression (QR). Results are sorted by quantile (*τ*), from 0.1 (A) to 0.9 (I). The solid and dashed lines show the Bonferroni-adjusted thresholds of 3.88 and 4.58 for alpha equals to 5% and 1%, respectively.
(TIFF)

S7 Fig Manhattan plot for days to end of flowering (DEF) using quantile regression (QR). Results are sorted by quantile (τ), from 0.1 (A) to 0.9 (I). The solid and dashed lines show the Bonferroni-adjusted thresholds of 3.88 and 4.58 for alpha equals to 5% and 1%, respectively.
(TIFF)
