# Supplementary material for: Quantile regression for genome-wide association study of flowering time-related traits in common bean
Source: PLoS One. 2018 Jan 4;13(1):e0190303. doi: 10.1371/journal.pone.0190303 (PMC5754186; doi:10.1371/journal.pone.0190303)
Supplement: S1 Table — (PDF) [file pone.0190303.s001.pdf]

S1 Table. Name of cultivars, year of release and research institution in charge of the 80 common bean cultivars.

Cultivars	Release	Institution	Cultivars	Release	Institution
Rico 23	1960	UFV	Pérola	1996	Embrapa
Carioca 1030	1970	IAC	IAPAR 81	1997	IAPAR
Rio Tibagi	1971	UFV	IPR Uirapurú	2000	IAPAR
Capixaba Precoce	1980	EMCAPA	BRS Valente	2001	Embrapa
Carioca 80	1980	IAC	BRSMG Talismã	2002	UFV/UFLA
Moruna	1980	IAC	IPR Graúna	2002	IAPAR
Iraí	1981	IPAGRO	BRS Campeiro	2003	Embrapa
Iapar 8 - Rio Negro	1983	IAPAR	BRS Grafite	2003	Embrapa
Milionário 1732	1983	EPAMIG	BRS Pontal	2003	Embrapa
Rico 1735	1983	EPAMIG	BRS Requinte	2003	Embrapa
BR - 2 Grande Rio	1985	PESAGRO	BRS Majestoso	2004	Embrapa
BR - 3 Ipanema	1985	PESAGRO	BRS Supremo	2004	Embrapa
BR1 - Xodó	1985	PESAGRO	IPR Colibri	2004	IAPAR
FT 120	1986	FT-sementes	IPR Saracura	2004	IAPAR
IAPAR 16	1986	IAPAR	SCS Guará	2004	EPAGRI
IAC Carioca	1987	IAC	BRSMG Pioneiro	2005	Embrapa
IAPAR 20	1987	IAPAR	IAC Tunã	2005	IAC
Rio doce	1987	EMCAPA	IAC Votuporanga	2005	IAC
BR-IPAGRO 2-Pampa	1989	EMCAPA	IAC-Apuã	2005	IAC
BR-IPAGRO1-Macanudo	1989	IPAGRO	IAC-Ybaté	2005	IAC
Carioca 1070	1989	IAC	BRS Cometa	2006	Embrapa
BR 6 - Barriga verde	1990	EMPASC	BRS Esplendor	2006	Embrapa
IAPAR 44	1990	IAPAR	IPR Eldorado	2006	IAPAR
Preto Uberabinha	1990	IPEACO/MG	IPR Gralha	2006	IAPAR
Diamante Negro	1991	Embrapa	IPR Tiziu	2006	IAPAR
IAPAR 31	1991	IAPAR	BRS Expedito	2007	Embrapa
Varre - Sai	1991	PSAGRO/RJ	IAC Alvorada	2007	IAC
Aporé	1992	EMGOPA	IPR 139	2007	IAPAR
BR - IPA 10	1992	IPA	IPR Tangará	2008	IAPAR
FT bonito	1992	FT- sementes	BRS Estilo	2010	Embrapa
IAPAR 57	1992	IAPAR	IPR Tuiuiú	2010	IAPAR

Onix	1992	EMGOPA	BRS Notável	2011	Embrapa
Ouro Negro	1992	Embrapa	IAC Formoso	2011	IAC
IAPAR 65	1993	IAPAR	IPR Campos Gerais	2011	IAPAR
Xamego	1993	EMGOPA	BRSMG Madrepérola	2012	UFV
BR- IPA 11-Brígida	1994	IPA/PE	IAC Imperador	2013	IAC
IAC - Carioca Pyatã	1994	IAC	IPR Andorinha	2013	IAPAR
IAC - Una	1994	IAC	VC 15	2013	UFV
Rudá	1994	EMCAPA	VP 22	2013	UFV
IAC - Carioca Akytá	1996	IAC	VP 33	2013	UFV
