# Supplementary figures and images for: Quantile regression for genome-wide association study of flowering time-related traits in common bean
Source: PLoS One. 2018 Jan 4;13(1):e0190303. doi: 10.1371/journal.pone.0190303 (PMC5754186; doi:10.1371/journal.pone.0190303)

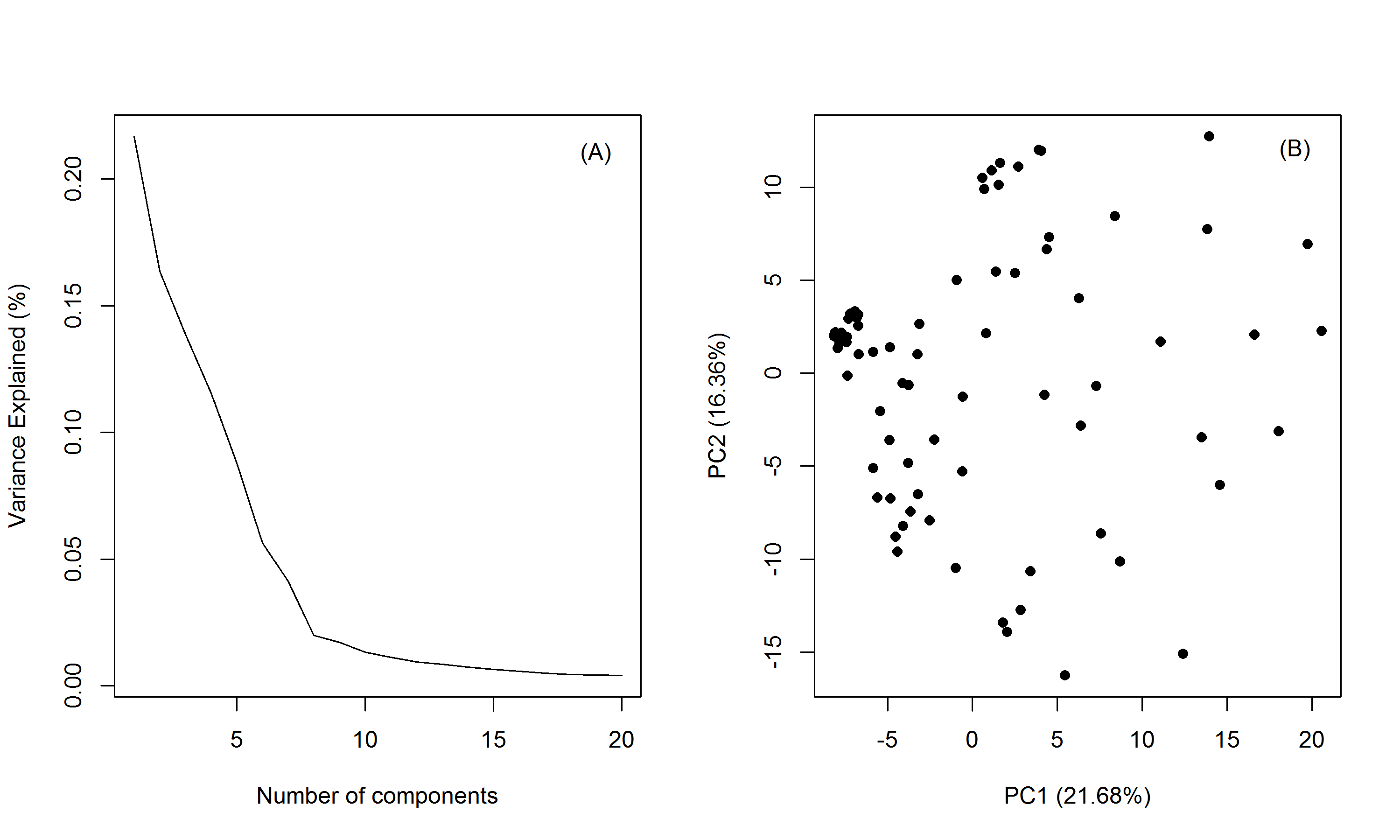

Supplement: S1 Fig — (A) Scree plot and variance explained (%) by each component. (B) Plot for PC1 against PC2 illustrating the population structure of 80 common bean genotypes. (TIFF) [file pone.0190303.s002.tiff]

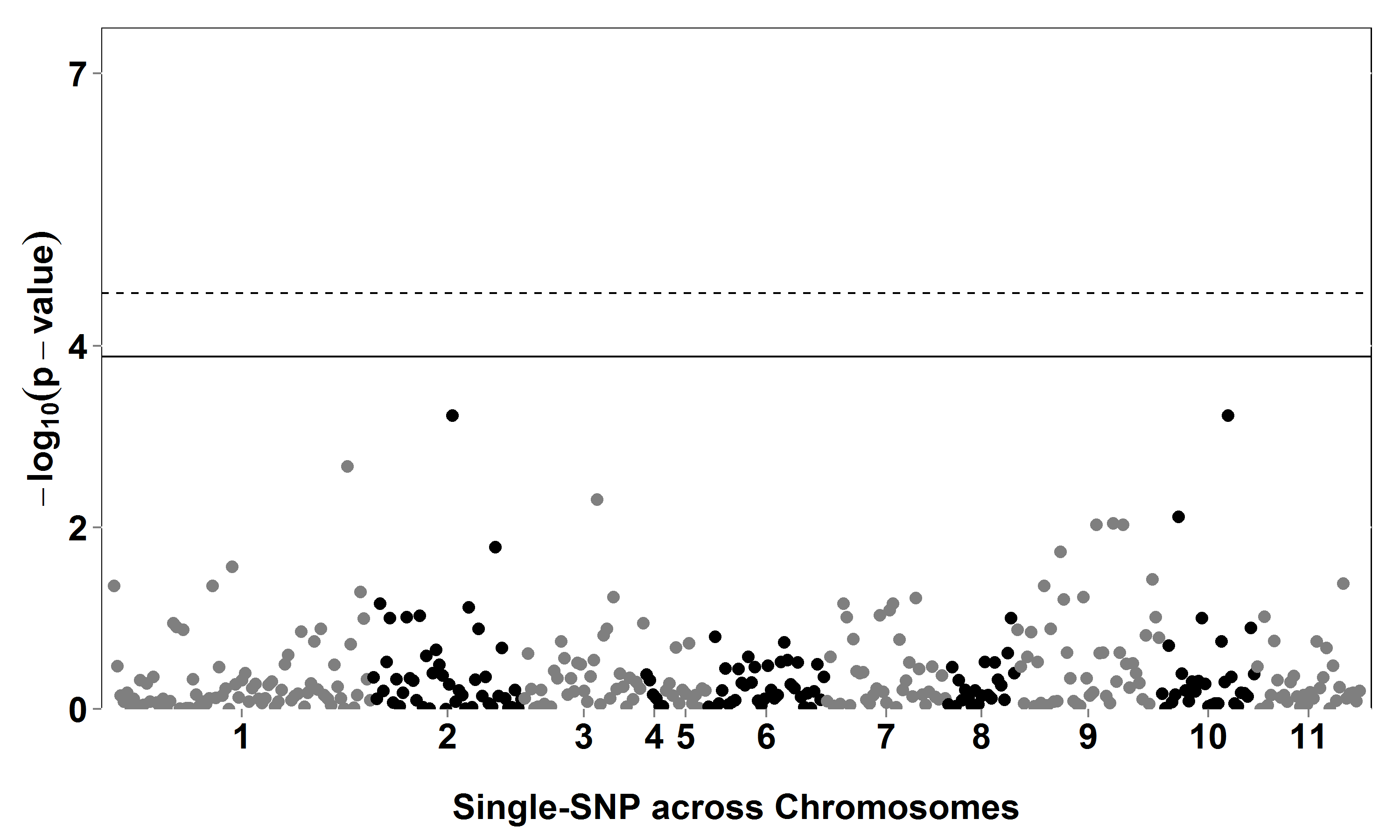

Supplement: S2 Fig — The solid and dashed lines show the Bonferroni-adjusted thresholds of 3.88 and 4.58 for alpha equals to 5% and 1%, respectively. (TIFF) [file pone.0190303.s003.tiff]

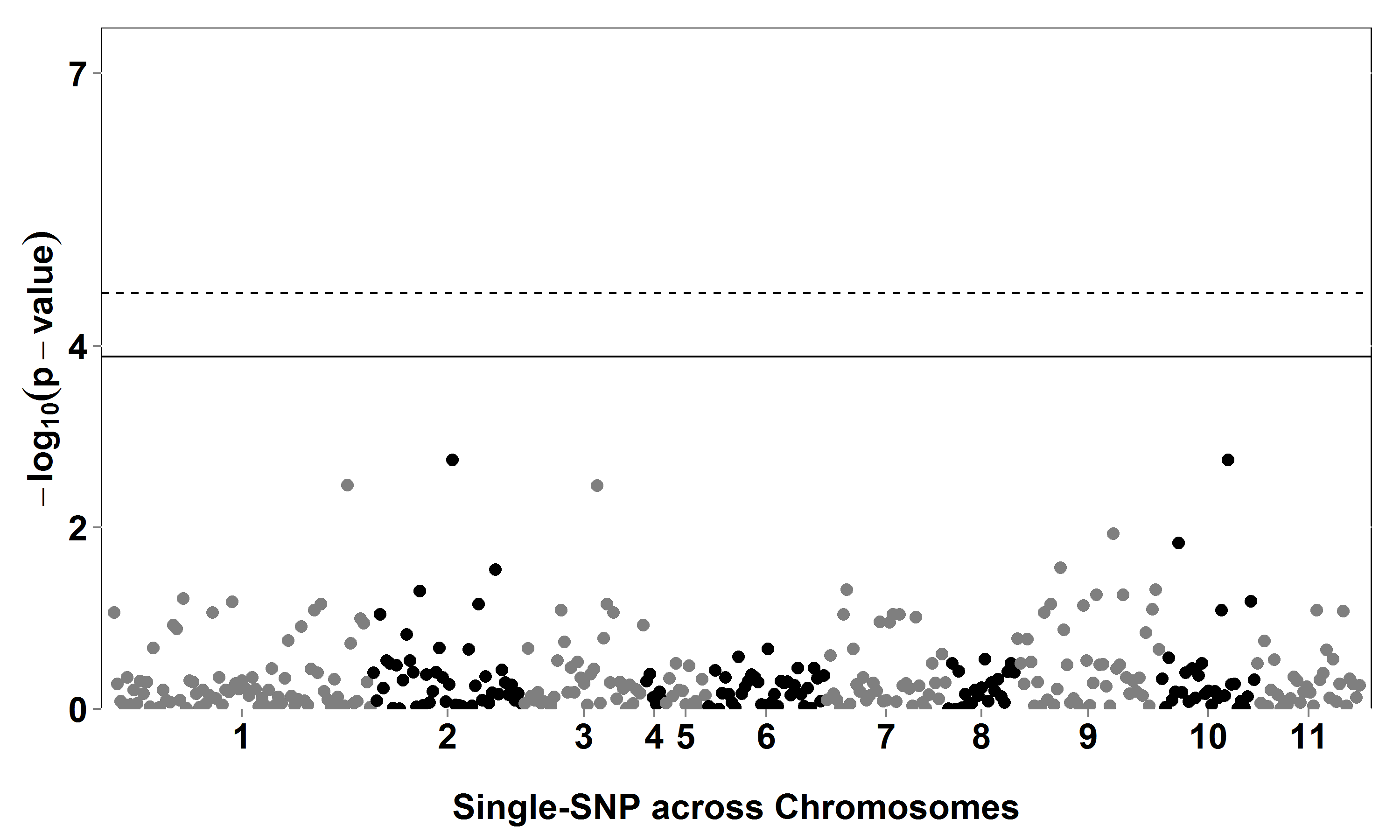

Supplement: S3 Fig — The solid and dashed lines show the Bonferroni-adjusted thresholds of 3.88 and 4.58 for alpha equals to 5% and 1%, respectively. (TIFF) [file pone.0190303.s004.tiff]

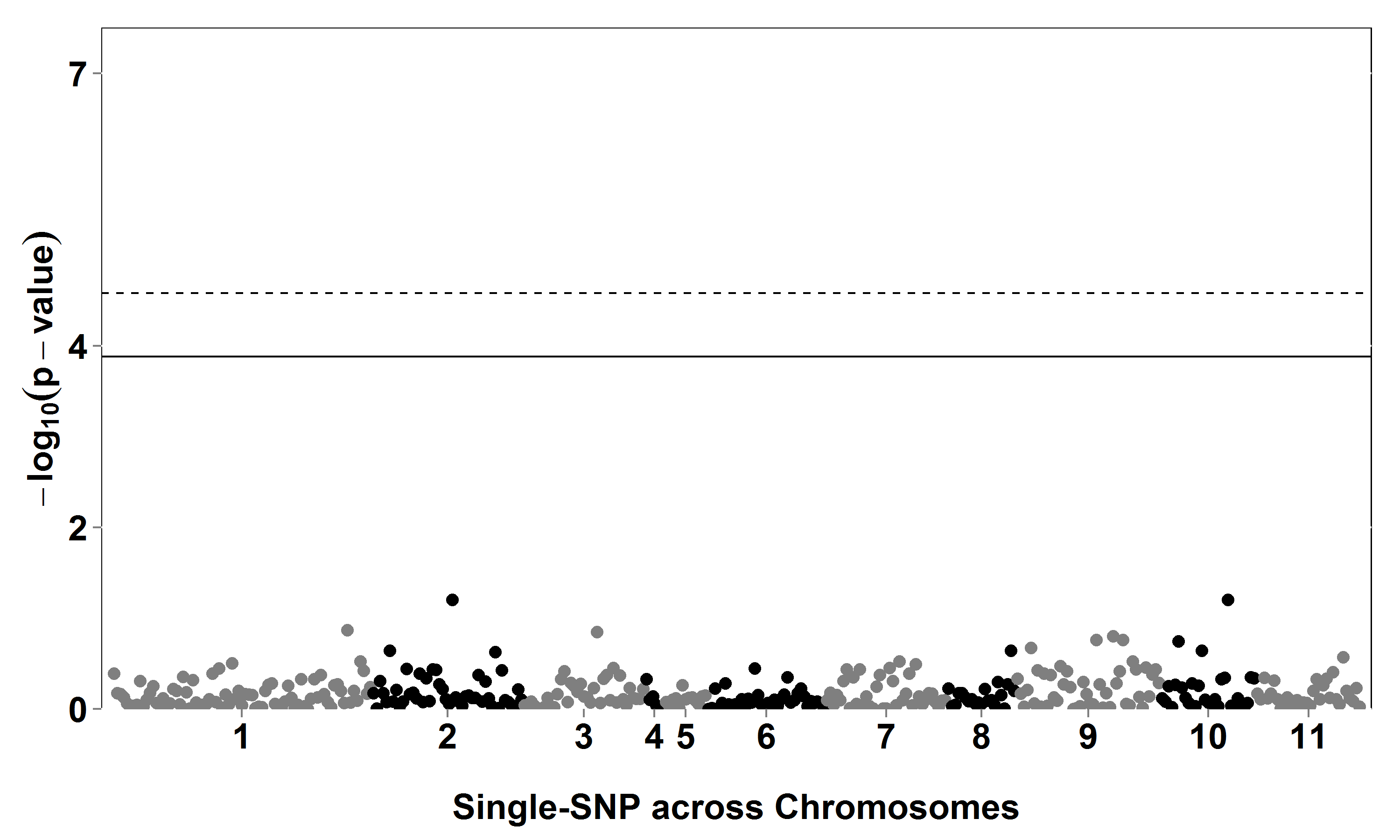

Supplement: S4 Fig — The solid and dashed lines show the Bonferroni-adjusted thresholds of 3.88 and 4.58 for alpha equals to 5% and 1%, respectively. (TIFF) [file pone.0190303.s005.tiff]

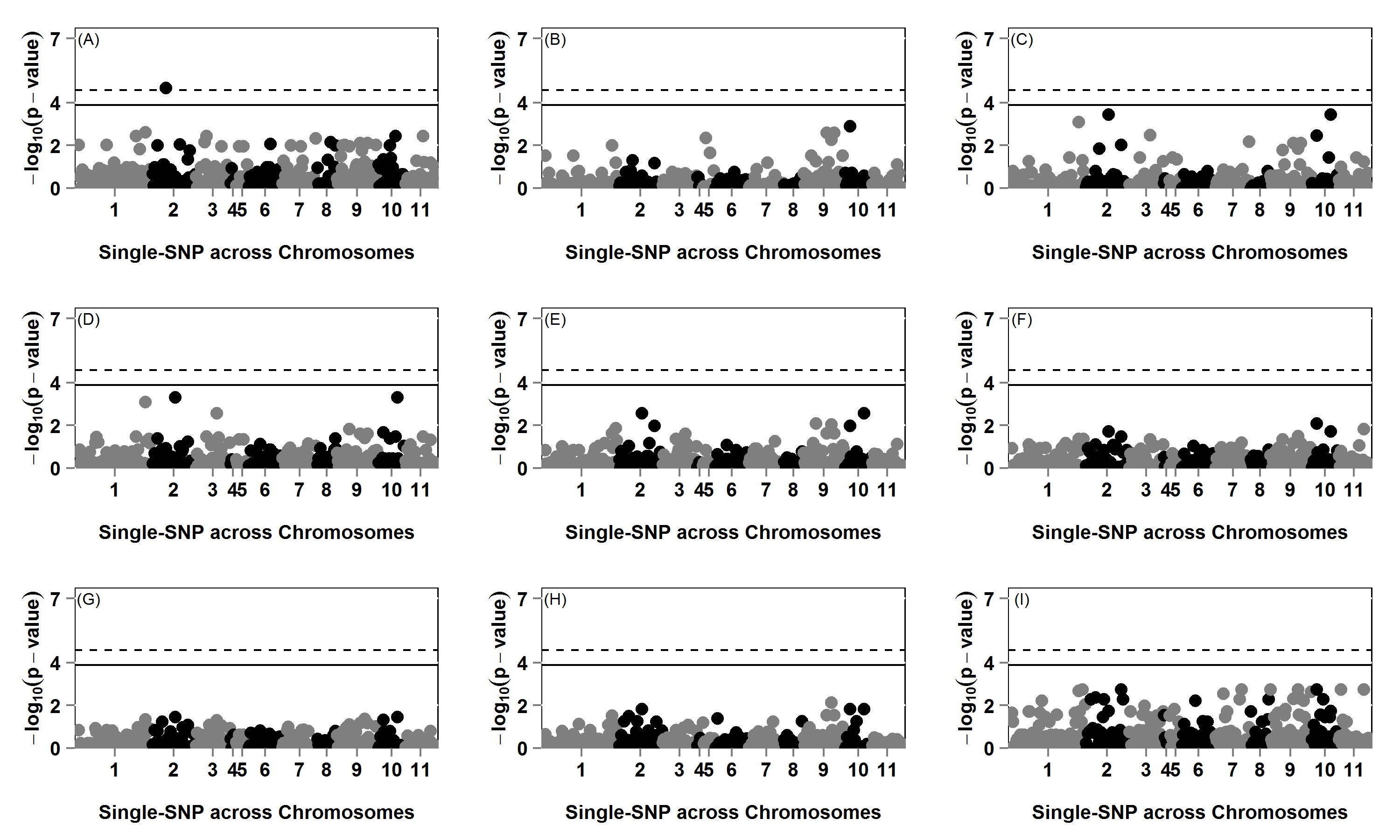

Supplement: S5 Fig — Results are sorted by quantile (τ), from 0.1 (A) to 0.9 (I). The solid and dashed lines show the Bonferroni-adjusted thresholds of 3.88 and 4.58 for alpha equals to 5% and 1%, respectively. (TIFF) [file pone.0190303.s006.tiff]

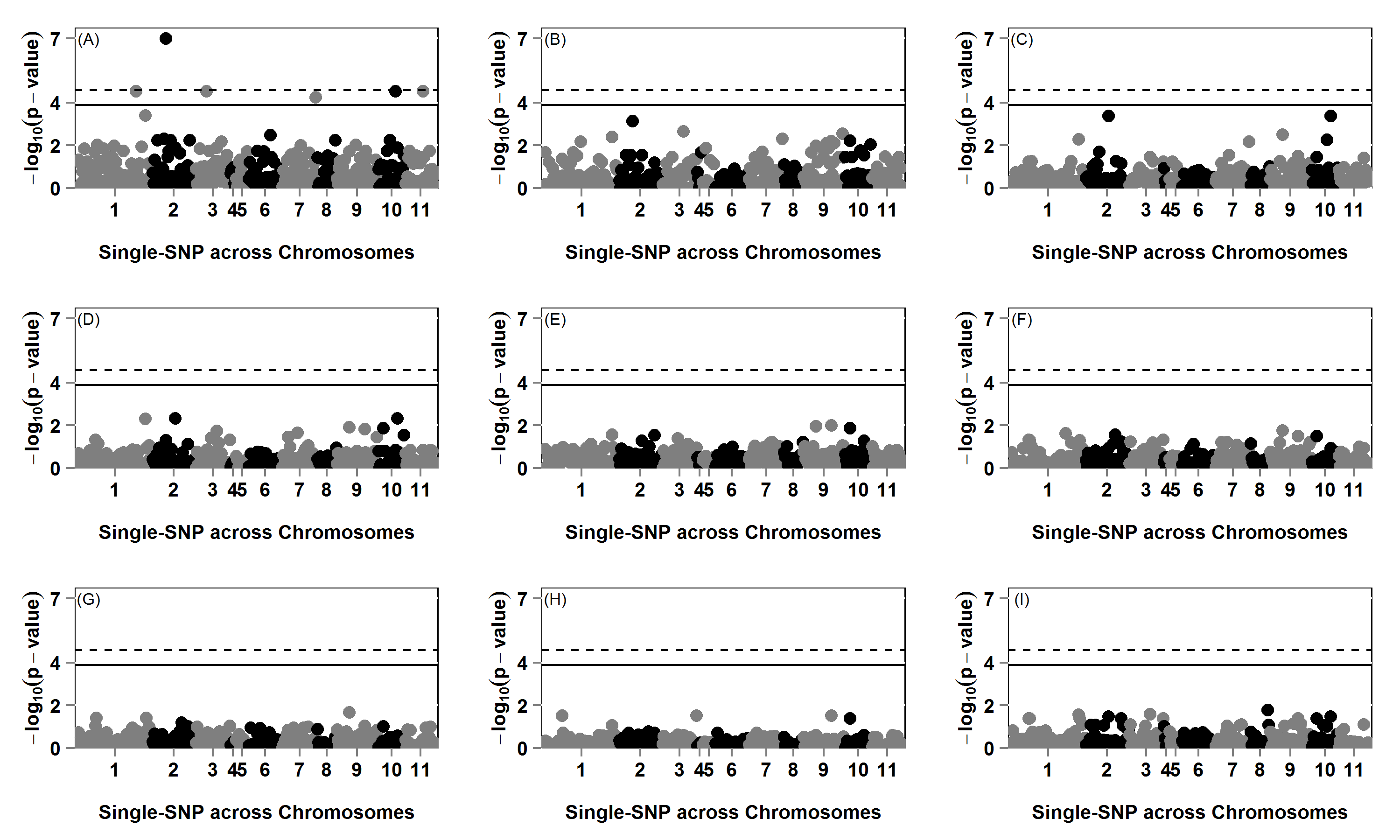

Supplement: S6 Fig — Results are sorted by quantile (τ), from 0.1 (A) to 0.9 (I). The solid and dashed lines show the Bonferroni-adjusted thresholds of 3.88 and 4.58 for alpha equals to 5% and 1%, respectively. (TIFF) [file pone.0190303.s007.tiff]

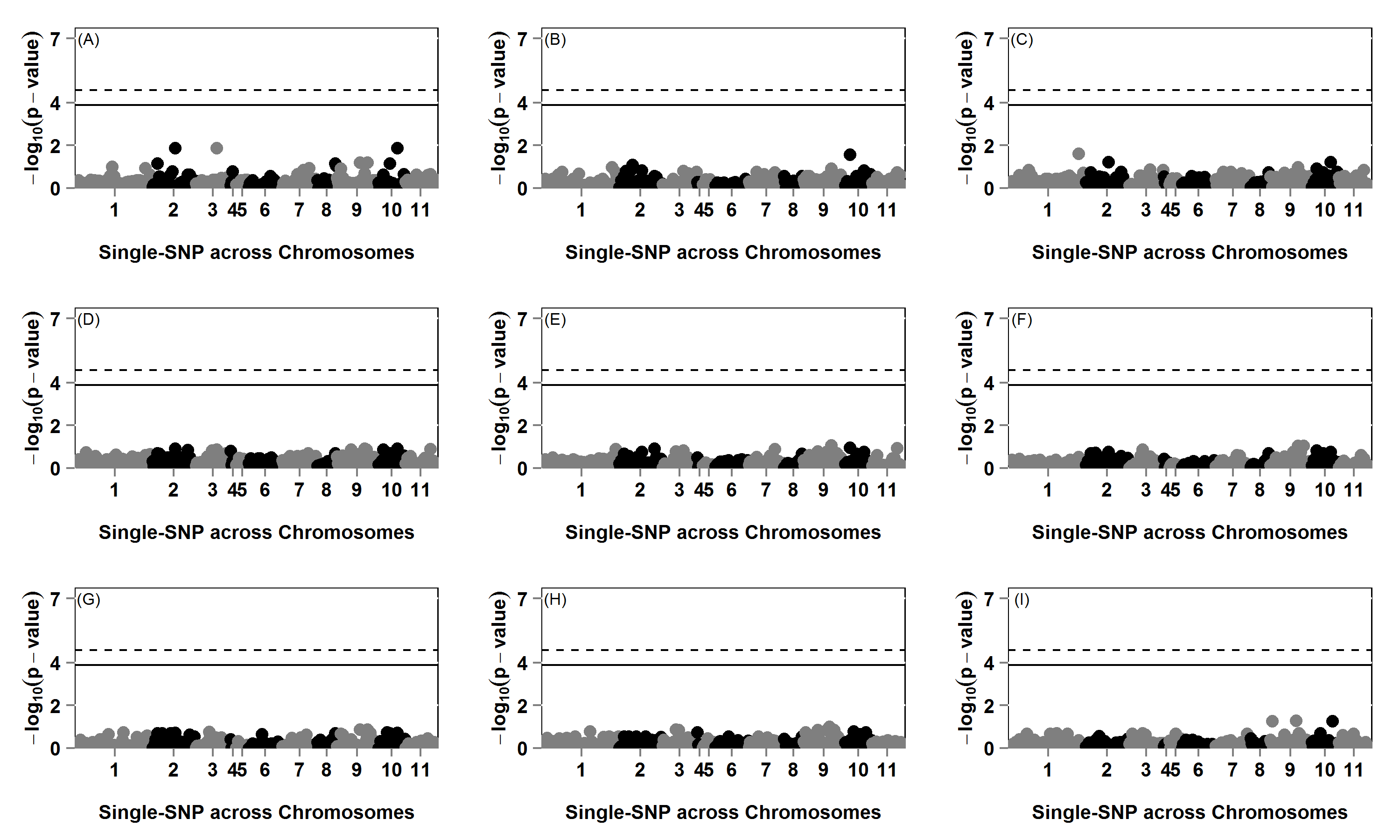

Supplement: S7 Fig — Results are sorted by quantile (τ), from 0.1 (A) to 0.9 (I). The solid and dashed lines show the Bonferroni-adjusted thresholds of 3.88 and 4.58 for alpha equals to 5% and 1%, respectively. (TIFF) [file pone.0190303.s008.tiff]
